# Effect of spectral degradation on speech intelligibility and cortical representation

**DOI:** 10.3389/fnins.2024.1368641

**Published:** 2024-04-05

**Authors:** Hyo Jung Choi, Jeong-Sug Kyong, Jong Ho Won, Hyun Joon Shim

**Affiliations:** ^1^Department of Otorhinolaryngology-Head and Neck Surgery, Nowon Eulji Medical Center, Eulji University School of Medicine, Seoul, Republic of Korea; ^2^Eulji Tinnitus and Hearing Research Institute, Nowon Eulji Medical Center, Seoul, Republic of Korea; ^3^Sensory-Organ Research Institute, Medical Research Center, Seoul National University School of Medicine, Seoul, Republic of Korea; ^4^Department of Radiology, Konkuk University Medical Center, Seoul, Republic of Korea; ^5^Hyman, Phelps and McNamara, P.C., Washington, DC, United States

**Keywords:** speech intelligibility, spectral degradation, vocoder, event-related potential, N2 and P3b

## Abstract

Noise-vocoded speech has long been used to investigate how acoustic cues affect speech understanding. Studies indicate that reducing the number of spectral channel bands diminishes speech intelligibility. Despite previous studies examining the channel band effect using earlier event-related potential (ERP) components, such as P1, N1, and P2, a clear consensus or understanding remains elusive. Given our hypothesis that spectral degradation affects higher-order processing of speech understanding beyond mere perception, we aimed to objectively measure differences in higher-order abilities to discriminate or interpret meaning. Using an oddball paradigm with speech stimuli, we examined how neural signals correlate with the evaluation of speech stimuli based on the number of channel bands measuring N2 and P3b components. In 20 young participants with normal hearing, we measured speech intelligibility and N2 and P3b responses using a one-syllable task paradigm with animal and non-animal stimuli across four vocoder conditions with 4, 8, 16, or 32 channel bands. Behavioral data from word repetition clearly affected the number of channel bands, and all pairs were significantly different (*p* < 0.001). We also observed significant effects of the number of channels on the peak amplitude [*F*_(2.006, 38.117)_ = 9.077, *p* < 0.001] and peak latency [*F*_(3, 57)_ = 26.642, *p* < 0.001] of the N2 component. Similarly, the P3b component showed significant main effects of the number of channel bands on the peak amplitude [*F*_(2.231, 42.391)_ = 13.045, *p* < 0.001] and peak latency [*F*_(3, 57)_ = 2.968, *p* = 0.039]. In summary, our findings provide compelling evidence that spectral channel bands profoundly influence cortical speech processing, as reflected in the N2 and P3b components, a higher-order cognitive process. We conclude that spectrally degraded one-syllable speech primarily affects cortical responses during semantic integration.

## 1 Introduction

Spectral degradation of speech limits successful comprehension. Informational or energetic noise can mask speech by degrading spectral information and thus reduce its intelligibility. Noise-vocoded speech is a form of spectrally degraded speech with distortion that was developed by Shannon et al. to simulate speech heard through a cochlear implant (CI) device (Shannon et al., 1995). The basic principle of vocoder processing is to decompose a speech signal into multiple bands, extracting the envelope waveform for each band modulating a carrier signal and then summate all amplitude-modulated carrier signals (Shannon et al., 1995; Dorman et al., 1997). Until now, CIs have comprised at most 40 channels (Advanced Bionics recently released implants with 120 spectral bands), and the technical limitations prevent a literal copy of the human cochlear, which can detect thousands of tonotopy resolutions across the range of 20–20,000 Hz. Accordingly, CI users suffer from hearing spectrally degraded speech.

Numerous studies that measured speech intelligibility consistently observed a clear stepwise increase in the number of correctly reported words with increasing number of channel bands in noise-vocoded speech (Hervais-Adelman et al., 2008; Souza and Rosen, 2009). Along with these behavioral data, the effect of number of channel bands on spectral information was also observed in several functional MRI studies. Increased intelligibility led to an increased percent change in the blood oxygen level-dependent signal in the temporal lobe (Davis and Johnsrude, 2003; Obleser et al., 2007; Evans et al., 2014).

While several electroencephalography (EEG) studies have replicated the influence of channel bands on cortical potentials, the current body of evidence is insufficient to draw definitive conclusions. Despite previous studies examining the channel band effect using earlier event-related potentials (ERP) components, such as P1, N1, and P2, a clear consensus or understanding remains elusive. In the study of Friesen et al. (2009), the effects of channel bands were reflected in the P1, N1, and P2 components in conditions involving 2, 4, 8, 12, and 16 channels, as well as ordinary speech. The study revealed that, as the number of channels of acoustic information increased, the peak amplitude of the neural response increased. In contrast, a recent study found inconsistent changes in the P1, N1, and P2 components when listening to vocoded speech on 4 and 22 channels (Dong and Gai, 2021). In another study, the type of vocoder carrier was shown to affect the neural responses; the responses to noise-vocoded stimuli were smaller and slower when measured using mismatch negativity compared with the responses to tone-vocoded stimuli (Xu et al., 2019). The P1, N1, and P2 components assessed in those studies focused on the neural responses derived from the physical or acoustic perception of speech. However, listening to speech with sparse information increases the compensatory reliance on top-down cognitive processes (Pals et al., 2020). A contribution of the frontal lobe was also evident, and the frontal operculum showed an elevated response to noise-vocoded speech (Davis and Johnsrude, 2003). Inconsistent findings and limitations in capturing higher-level cognitive processes for the earlier ERPs prompted a shift in focus to the N2 and P3 components in our study, rather than the P1, N1, and P2 components, as cortical potentials to replicate the channel band effect. We hypothesized that the N2 and P3 components, associated with lexical information assessment and stimulus categorization, would offer a more detailed exploration of the channel band effect in noise-vocoded speech.

N2 and P3 deflections are elicited in response to task-relevant stimuli in an oddball paradigm (Luck, 2014). The N2 component refers to a frontocentral negativity occurring 200 to 350 ms after stimuli (Schmitt et al., 2000; Folstein and Van Petten, 2008). The latency, however, is typically delayed in tasks with complex stimuli, such as speech words, encompassing a broader time window (350 to 800 ms), depending on task difficulty or hearing condition. Thus, this prolonged N2 is often referred to as N2N4 (Voola et al., 2023), reflecting cortical access to lexical information and semantic categorization in the deaf population (Finke et al., 2016). Both N2 and N4 are cortical responses related to the lexical selection process, analogous to the functional interpretation of the N400 component (Van den Brink and Hagoort, 2004). P3 has subcomponents of P3a and P3b. Unlike P3a, P3b reflects the effortful allocation of resources to discriminate and interpret auditory stimuli (Volpe et al., 2007; Voola et al., 2022). The P3b component is also associated with updating working memory, and prolonged latencies may be interpreted as slower stimulus evaluation (Beynon et al., 2005; Henkin et al., 2015). The P3b component is maximally observed parietally, elicited by unpredictable and infrequent shifts. Any manipulation to delay stimulus categorization increases P3b latency and decreases amplitude (Johnson, 1988). Studies have shown that both N2 (Finke et al., 2016) and P3b (Beynon et al., 2005) were prolonged in CI users compared to normal hearing listeners, implicating a slower stimulus evaluation in CI users due to device limitations such as poor spectral density. However, there is no report on the N2 and P3b regarding the effect of spectral manipulation in speech.

In the current study, we aimed to investigate differences in acoustic-based semantic processing in noise-vocoded speech. We employed a one-syllable oddball paradigm instead of sentences, which allowed us to: (1) minimize the redundancy of cues, (2) reduce top-down expectations in the context (Bae et al., 2022), and (3) control for individual differences in education and attention ability. Stimulus categorization, such as determining whether a stimulus is living or non-living, represents one of the simplest forms of higher-order processing in speech. We hypothesized that measuring the N2 and P3b components through the vocoded one-syllable oddball paradigm could robustly evaluate the impact of spectral degradation on acoustic-based semantic processing. The purpose of this study was to objectively assess the effect of channel bands, focusing on higher-level cognition such as lexical information assessment and stimulus categorization.

## 2 Subjects and methods

### 2.1 Subjects

The main experiment’s sample size was determined through a pilot study with four participants. The effect size (*ηp^2^*) was obtained through a pilot study and using G*Power software (latest version 3.1.9.7; Heinrich-Heine-Universität Düsseldorf, Düsseldorf, Germany; Faul et al., 2007), the recommended sample size of 17 was derived by inputting the effect size into the software. To account for potential dropouts (20%), we aimed to recruit 21 participants. Despite one withdrawal, data analysis was ultimately conducted with a total of 20 young adults with normal hearing (mean age: 29.8 ± 5.9 years old; women, 29.0 ± 6.5 years old; men, 30.6 ± 5.6 years; 10 males, 10 females).

The pure-tone average across 500, 1,000, 2,000, and 3,000 Hz was 7.7 [standard deviation (SD) =3.0] decibel (dB) hearing level (HL) on the right side and 6.8 (SD = 2.9) dB HL on the left side. Table 1 shows the detailed demographic information of the participants. The study was conducted in accordance with the Declaration of Helsinki and the recommendations of the Institutional Review Board of **** Medical Center. Written informed consent was obtained from all subjects. After subjects signed the consent form, a copy was given to them.

**Table 1:** Demographic summary of the participants.

No.	Sex	Age (years)	Handedness	Education (years)	Pure tone average (right, dB HL)	Pure tone average (left, dB HL)
1	M	38	R	18	8.8	8.8
2	F	34	R	12	10.0	10.0
3	M	36	R	16	8.8	10.0
4	M	24	R	16	8.8	6.3
5	M	31	R	16	7.5	8.8
6	F	20	R	12	5.0	5.0
7	F	21	R	12	5.0	5.0
8	M	36	R	16	5.0	5.0
9	F	27	R	14	6.3	8.8
10	M	26	R	14	8.8	3.8
11	M	23	R	12	11.3	11.3
12	M	36	R	18	2.5	6.3
13	F	27	R	18	3.8	2.5
14	F	32	R	16	10.0	7.5
15	M	29	R	16	11.3	7.5
16	F	23	R	16	6.3	0.0
17	F	39	R	14	5.0	5.0
18	F	32	R	16	15.0	11.3
19	M	27	R	16	7.5	7.5
20	F	35	R	18	7.5	5.0
Mean		29.8		15.3	7.7	6.8
SD		5.9		2.1	3.0	2.9

The pure-tone average was calculated as the average of the thresholds at frequencies of 500, 1,000, 2,000, and 3,000 Hz.

### 2.2 Stimuli

Stimuli were recorded by a female speaker reading five lists of 25 monosyllable animal or non-animal Korean words using a lapel microphone (BY-WMA4 PRO K3, BOYA, Shenzhen, Hong Kong) in a soundproof booth. All the recorded stimuli were sampled at a rate of 44,100 Hz, and the overall root mean square amplitude was set at −22 dB. The phonetic balance, an equal range of the phonetic composition of speech, words in common usage, and familiarity with the words were considered when the word lists were chosen. Based on the frequency of occurrence of conversational sounds, we created a CVC word set with 19 initial consonants, 21 vowels, and 7 final consonants.

The equivalent average difficulty and phoneme composition of the lists were verified. The long-term average speech spectrum of the recorded syllable was analyzed using Computerized Speech Lab (CSL model 4500b, KayPENTAX Elemetrics Corporation, Lincoln Park, NJ, United States).

**Figure 1:**
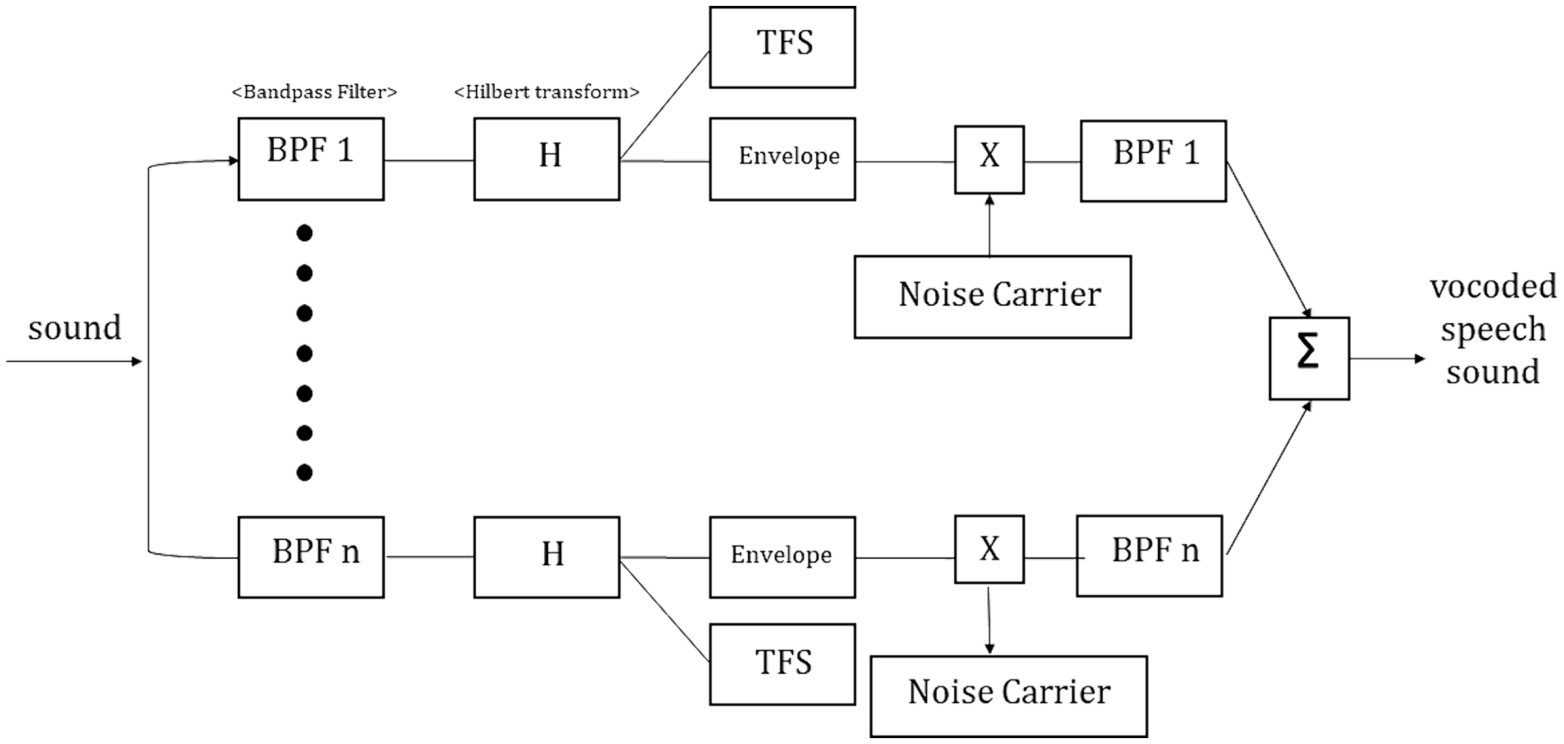
Illustration depicting the generation of the noise-vocoded signal. The input signals were band-pass filtered into 4 (BPF1), 8 (BPF2), 16 (BPH3), and 32 (BPF4) channel bands prior to Hilbert transformation. After separating the envelopes from the temporal fine structures, the vocoder speech signal was generated by adding a noise carrier to the envelopes.

**Figure 2:**
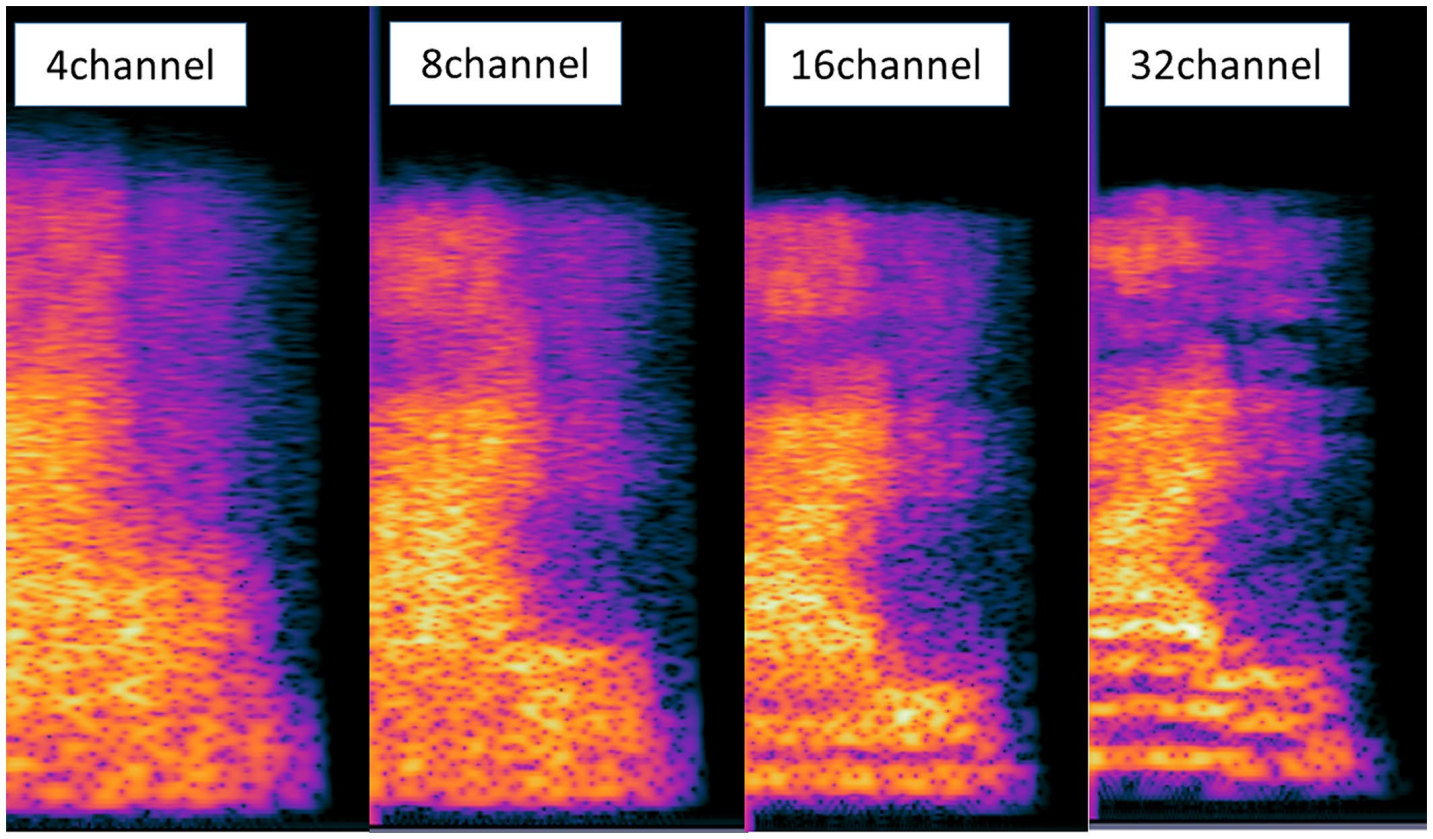
Spectrogram of the signals of the conditions with 4, 8, 16, and 32 channel bands. With fewer channel bands, the speech becomes more spectrally degraded and harder to understand. The information in the spectra was blurriest in the condition with 4 channel bands.

Noise-vocoding involves passing a speech signal through a filter bank to extract time-varying envelopes associated with the energy in each spectral channel band. The extracted envelopes were multiplied by white noise and combined after re-filtering (Shannon et al., 1995). First, the initial signal underwent processing through band-pass filtering, creating multiple channels (4, 8, 16, or 32 channels). The cut-off frequencies for each individual band-pass filter were determined using logarithmically spaced frequency bands, employing the Greenwood function (e.g., for 4 channels: [80, 424, 1,250, 3,234, and 8,000 Hz]). The center frequency of each channel was computed as the geometric mean between the two cutoff frequencies associated with that specific channel. The collective input frequency range spanned from 80 to 8,000 Hz. Subsequently, the amplitude envelope was extracted for each frequency band by means of half-wave rectification. Finally, we then summed the signals to generate the noise-vocoded speech session (Shannon et al., 1995; Faulkner et al., 2012; Evans et al., 2014). Vocoding was performed using a custom MATLAB script (2020a, MathWorks, Inc., Natick, MA, United States) using 4, 8, 16, or 32 spectral channels with a temporal envelope modulation cut-off frequency fixed at 500 Hz. Figure 1 illustrates the flow of generating noise-vocoded signal. Noise-vocoded speech sounds like a harsh whisper with only a weak sense of pitch. From our lab experience, speech synthesized with fewer than 4 bands were hard to understand. Resulting signals sound like a harsh whisper (Narain et al., 2003), and spectral detail decreases as the number of channel band decreases, as seen in Figure 2.

### 2.3 Procedure

#### 2.3.1 Behavioral test

The perception of one-syllable words was tested in four different channel band conditions (4, 8, 16, or 32 channel vocoder), using five lists, with each containing 25 Korean monosyllabic words. The participants were asked to repeat the words after they were presented through a loudspeaker placed 1 meter in front of them. The stimulus intensity was set to 70 dB sound pressure level (SPL) when calibrated at the listener’s head position, 1 meter away from the loudspeaker. In addition, during the recording of the N2 and P3b, we assessed participants’ accuracy in identifying one-syllable non-animal words from animal/non-animal word sets that were vocoded in four different channel band conditions (4, 8, 16, and 32 channel bands).

#### 2.3.2 EEG

The neural responses were recorded across 31 AG-Ag/Cl sintered electrodes placed according to the international 10–20 system (Jasper, 1958) in an elastic cap using the actiCHamp Brain Products recording system (BrainVision Recorder Professional, V.1.23.0001, Brain Products GmbH, Inc., Munich, Germany) while the participant sat in a dimly lit, sound-attenuated, electrically soundproof booth. Electro-oculogram and electrocardiogram were also tagged to trace eye movements and heartbeats. EEG data were digitized online at a sampling rate of 1,000 Hz. All 32 electrodes were referenced to the algebraic average of all electrodes/channels and were therefore unbiased to any electrode position. The ground electrode was placed between electrodes Fp1 and Fp2. Software filters were set at low (0.5 Hz) and high (70 Hz) cutoffs. A notch filter was set at 60 Hz to prevent powerline noise. The impedance of each scalp electrode was kept below 5 kΩ throughout the recording, as suggested by the manufacturer’s guide.

##### 2.3.2.1 Oddball paradigm

Based on a one-syllable task paradigm, the participants listened to animal words or non-animal but sensible words. Overall, 70% of the trials involved an animal target word (e.g., mouse, snake, bear; all monosyllabic in Korean). Sitting upright, the listeners were instructed that monosyllabic animal words were heard but they were to push the button otherwise as soon as possible. This controlled for the participant’s attentive level during recording, and this is for recording behavioral performance identifying non-animal words from animal words. The participants were randomly presented with six blocks of 210 animal words and 90 non-animal words in four channel band conditions, totaling 1,200 trials. The interstimulus interval was fixed in 2,000 ms, allowing for a jitter of 2–5 ms. The order of presentation was randomized within each block, and the order of blocks was counterbalanced among listeners using E-Prime software (version 3, Psychology Software Tools, Inc., Sharpsburg, PA). Each block was separated by a 2~5-min break. A familiarizing session ensured the participants understood the task and their muscles were sufficiently relaxed. The intensity of the sound was fixed approximately at 70 dB SPL when calibrated at the listener’s head position, 1 meter from the loudspeaker.

##### 2.3.2.2 Preprocessing of the neural signals

The data were preprocessed and analyzed using Brain Vision analyzer (version 2.0, Brain Products GmbH, Inc.) and MATLAB R2019b (MathWorks Inc.) with EEGLAB v2021 (Delorme and Makeig, 2004), and Fieldtrip (Oostenveld et al., 2011) toolboxes. The EEG was filtered with a 0.1 Hz high-pass filter (Butterworth with a 12 dB/octave roll-off) and low-pass filtered at 50 Hz (Butterworth with a 24 dB/octave roll-off). The first three trials were excluded from analyses. The data were resampled at 256 Hz. Independent component analysis (ICA) was used to reject artifacts associated with eye blinks and body movement (average 4 independent components, range 3–6) and reconstructed (Makeig et al., 1997; Jung et al., 2000), transforming to the average reference. EEG waveforms were then time-locked to each stimulus onset and segmented from 200 ms prior to the stimulus onset to 1,000 ms after the stimulus onset. Baseline correction was performed accordingly. Prior to averaging, bad channels were interpolated using a spherical spline function (Perrin et al., 1989), and segments with values greater than ±70 μV at any electrode were rejected. All participants had at least 180–200 out of 210 usable animal trials and 78–86 usable non-animal trials per vocoder channel-band condition. An average wave file was generated for each subject for each condition. Based on the grand average computed across all conditions and participants, latency ranges for N2 and P3b were determined according to the literature and the peak latency was measured using a half area quantification, which may be less affected by latency jitter (Luck, 2014; Finke et al., 2016). Difference waveforms were constructed based on the subtraction of target stimuli from standard stimuli within conditions (Deacon et al., 1991). The area latency and amplitude of the N2 and P3b difference waveforms at each condition were compared. The time windows for N2 and P3b analysis were defined from each average waveform. In our data, the time windows for N2 and P3b were set as 280–870 ms and 280–840 ms, respectively. N2 was measured by averaging the signals from the frontocentral electrodes (Fz, FC1, FC2, and Cz), while P3b was measured using the parietal electrodes (CP1, CP2, P3, P4, and Pz), as outlined in Finke et al. (2016).

### 2.4 Statistical analysis

Repeated-measures analysis of variance (RM ANOVA) was used to test the effects of vocoder channel-band on behavioral accuracy and N2 and P3b area peak amplitude and latency. Greenhouser–Geisser correction was applied to the statistical comparisons for which the Mauchly test indicated violation of sphericity. Follow-up *post hoc* Bonferroni corrected tests were also used. Significance was inferred for corrected *p*-values of <0.05. Correlation analyses were performed using Pearson’s correlation test. All statistical analyses were performed using IBM SPSS software (ver. 25.0; IBM Corp, Armonk, NY, United States) and the built-in functions in MATLAB (2014a, 2019a, MathWorks Inc.). Data are presented as the mean ± SD. Outliers were defined as values that differed from the mean by ±2 SD.

## 3 Results

### 3.1 Behavioral data

**Figure 3:**
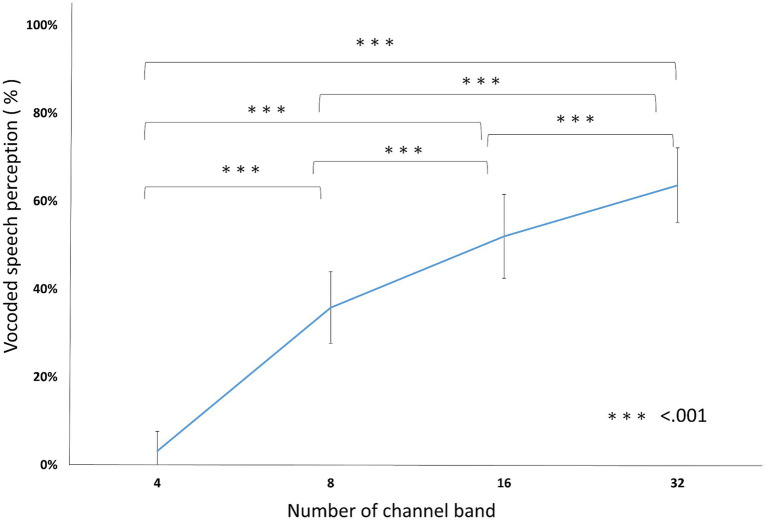
Vocoded speech perceptions. The conditions with fewer channel bands scored lower than conditions with more channel bands. All of the pairs were significantly different from each other. ^***^p < 0.001.

In vocoded speech perception, the accuracy was 3.20% ± 5.27, 35.80% ± 8.10, 52.00% ± 9.94, and 63.60% ± 9.88 in 4, 8, 16, and 32 channel band conditions, respectively. RM ANOVA showed a significant effect of the number of channel bands [*F*_(3, 57)_ = 284.70, *p* < 0.001, *ηp^2^* = 0.937]. All the pairs were significantly different (*P_Bonf_* < 0.001; Figure 3; Table 2).

**Table 2:** ANOVA table for vocoded speech perception.

	Sum of square	df	Mean square	F	*p*	ηp^2^
Channel	2581.938	3	860.646	284.697	<0.001	0.937
Residual	172.313	57	3.023			

### 3.2 Effects of spectral degradation on cortical representation

**Figure 4:**
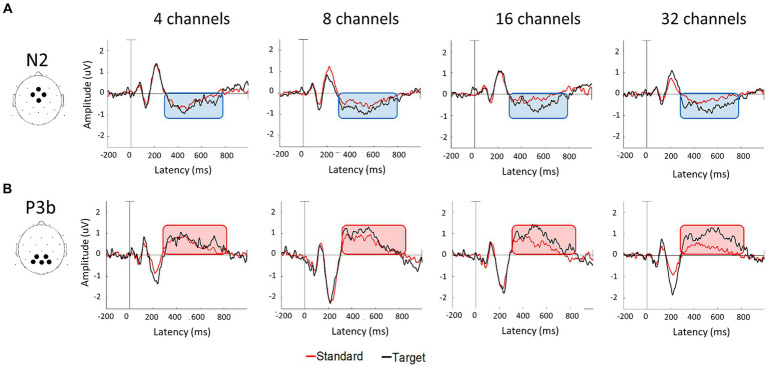
Grand average waveforms of N2 **(A)** and P3b **(B)** components at each number of channel conditions. N2 were measured by averaging the signals in the frontocentral electrodes (Fz, FC1, FC2, and Cz) and P3b were measured in the parietal electrodes (CP1, CP2, P3, P4, and Pz). The area time windows determined from the current data were 280–870 msec for N2 and 280–840 msec for P3b components.

**Figure 5:**
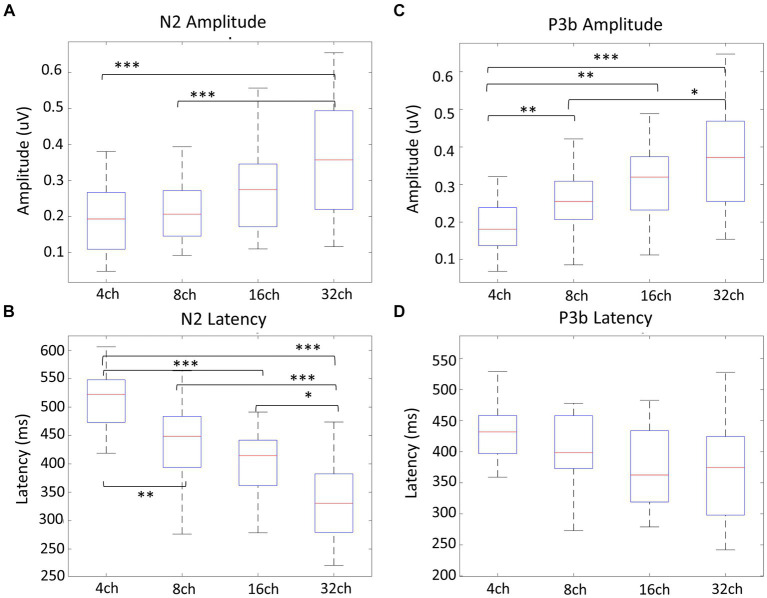
Amplitude and latency comparisons of N2 and P3b between channel bands. Regarding the N2 peak amplitude **(A)**, there was a significant difference between 4 and 8 channel bands compared to 32 channels (all *p* < 0.001), and for N2 peak latency **(B)**, all pairs showed significant differences (all *p* < 0.05). In terms of P3b amplitude comparisons, 4 channel bands significantly differed from all channels (all *p* < 0.01), while 8 channel bands differed from 32 channel bands (*p* = 0.010) **(C)**. All pairs showed no significant differences for P3b latency **(D)**. ^*^p < 0.05, ^**^p < 0.01, ^***^p < 0.001.

Figure 4A displays the grand-averaged waveforms of the N2 component with waveforms elicited by standard stimuli (animal) in red and target (non-animal) in black. Figure 4B illustrates the grand-averaged waveforms and the difference between animal and non-animal stimuli for the P3b component. Shades represent the areas of N2 (light blue) and P3b (pink). RM ANOVA (four channel-band conditions) for N2 showed significant main effects of the number of channel bands on peak amplitude [*F*_(2.006, 38.117)_ = 9.077, *p* < 0.001, *ηp^2^* = 0.323] and peak latency [*F*_(3, 57)_ = 26.642, *p* < 0.001, *ηp^2^* = 0.584; Figure 4A; Table 3]. In the comparisons of N2 peak amplitude based on the number of channel bands shown in Figure 5A, significant differences were observed between the conditions with 4 and 8 channel bands and the condition with 32 channels (all *P_Bonf_* < 0.001). The peak amplitudes were 0.19 ± 0.10, 0.21 ± 0.08, 0.30 ± 0.20, and 0.37 ± 0.16 μV for the conditions with 4, 8, 16, and 32 channel bands, respectively. Regarding the comparisons of N2 peak latency, as illustrated in Figure 5B, all pairs were significantly different from each other (all *P_Bonf_* < 0.05) except for the conditions with 8 vs. 16 channel bands. The peak latencies were 504.38 ± 63.43, 435.06 ± 77.44, 398.75 ± 63.12, ad 331.50 ± 73.35 ms for the conditions with 4, 8, 16, and 32 channel bands, respectively.

**Table 3:** ANOVA table for the amplitude and latency in N2.

		Sum of square	df	Mean square	F	*p*	ηp^2^
Amplitude	Channel	0.397	2.006	0.198	9.077	<0.001	0.323
	Residual	0.831	38.117	0.022			
Latency	Channel	312064.902	3	104021.634	26.642	<0.001	0.584
	Residual	222549.551	57	3904.378			

RM ANOVA (four channel-band conditions) for P3b difference showed significant main effects of the number of channel bands on peak amplitude [*F*_(2.231, 42.391)_ = 13.045, *p* < 0.001, *ηp^2^* = 0.407] and peak latency [*F*_(3, 57)_ = 2.968, *p* = 0.039, *ηp^2^* = 0.135; Figure 4B; Table 4]. Regarding the comparisons of P3b amplitude in terms of the number of channel bands shown in Figure 5C, the condition with 4 channel bands differed significantly from the condition with all channels (all *P_Bonf_* < 0.01), and the condition with 8 channel bands differed from the condition with 32 channel bands (*P_Bonf_* = 0.010). The peak amplitudes of P3b were 0.19 ± 0.08, 0.26 ± 0.08, 0.31 ± 0.11, and 0.37 ± 0.14 μV for the conditions with 4, 8, 16, and 32 channel bands, respectively. There were no significant differences in pairs in post-hoc comparisons for P3b latency. The peak latencies of P3b were 422.80 ± 76.35, 403.05 ± 59.41, 373.20 ± 64.07, and 363.75 ± 77.57 ms for the conditions with 4, 8, 16, and 32 channel bands, respectively (Figure 5D).

**Table 4:** ANOVA table for the amplitude and latency in P3b.

		Sum of square	df	Mean square	F	*p*	ηp^2^
Amplitude	Channel	0.343	2.231	0.154	13.045	<0.001	0.407
	Residual	0.499	42.391	0.012			
Latency	Channel	44309.700	3	14769.900	2.968	0.039	0.135
	Residual	283689.800	57	4977.014			

### 3.3 Correlation of neural response with behavioral data

**Figure 6:**
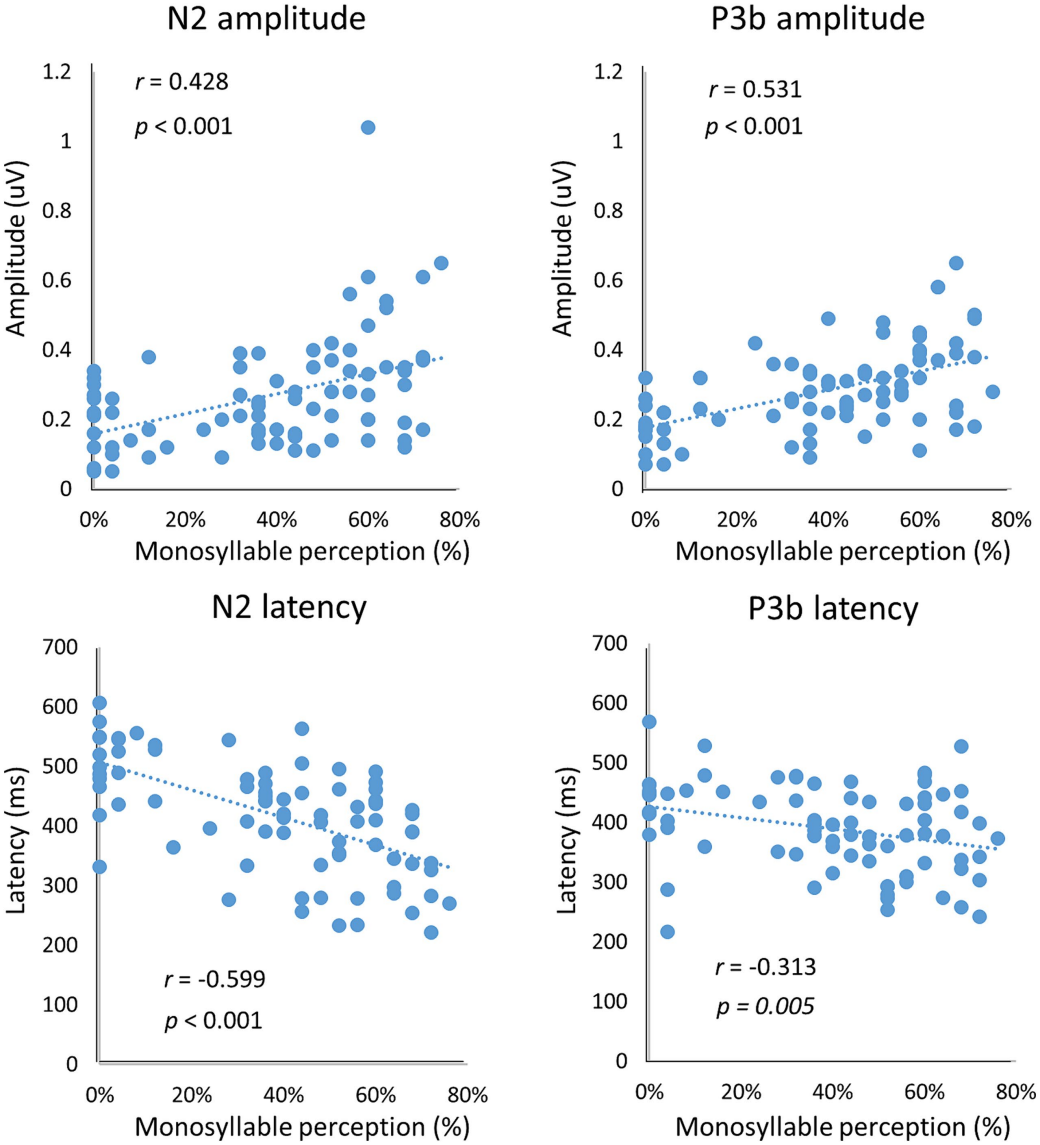
Correlation of neural response with behavioral data. There were significant correlations between the behavioral responses and N2 and P3b peak amplitudes/latencies (all *p* < 0.01).

We also determined correlations between vocoded speech perception and the neural response of N2 and P3b in terms of latency and amplitude. Significant correlations were found between the N2 peak amplitude/latency and the behavioral accuracy in vocoded speech perception (amplitude: *r* = 0.428, *p* < 0.001; latency: *r* = −0.599, *p* < 0.001; Figure 6). Similarly, there were significant correlations between the P3b peak amplitude/latency and the behavioral accuracy in vocoded speech perception (amplitude: *r* = 0.531, *p* < 0.001; latency: *r* = −0.313, *p* = 0.005; Figure 6).

## 4 Discussion

We found slowed N2 and P3b latencies, indicating delayed access to lexical information and semantic categorization in a small number of channel band conditions. Although neural responses were not significantly different in all pairs of channel bands, there was a consistent pattern of increasing amplitude and decreasing latency as the number of channel bands increased, aligning with the behavioral results. Behavioral tests indicated, as expected, that increasing the number of channel bands led to a stepwise improvement in the test of intelligibility, with all pairs showing significant differences. In addition, we found a strong correlation between N2 and P3b peak amplitudes/latencies and behavioral accuracy. This supports the effective representation of the channel band effect in a noise vocoder on N2 and P3b, confirming our hypothesis that spectral degradation with a noise vocoder significantly influences the neural representation of semantic processing.

Some studies showed the vocoder channel effect on P1-N1-P2 responses using CVC tokens (Friesen et al., 2009) or the DISH-DITCH continuum (Anderson et al., 2020). However, these studies compared the vocoded condition with the unprocessed condition and did not involve channel-specific multiple comparisons as in our current study. Moreover, a recent study found inconsistent changes in the P1, N1, and P2 components of ERP when listening to vocoded speech on 4 and 22 channels (Dong and Gai, 2021).

Other studies have examined later ERPs (>300 ms post-stimuli) to assess cognitive spare capacity for measuring channel band effect (Strauss et al., 2013; Banellis et al., 2020; Hunter, 2020). Banellis et al. (2020) investigated the role of the P3a component and the impact of top-down expectations in a word-pair priming task involving degraded (noise-vocoded) speech. The findings suggest that expectations play a crucial role in the comprehension of degraded speech, influencing neural responses. Hunter (2020) explored cognitive demand during listening to noise-vocoded spoken sentences, analyzing the impact of cognitive (memory) load and sentence predictability on electrophysiological measures, specifically the P300/late positive complex and N400. Strauss et al. (2013) measured the N400 evoked by a congruent—incongruent final object in a sentence paradigm using vocoders. In the 8-band condition, the N400 amplitude was attenuated, and the peak was significantly delayed by approximately 78 ms compared with clean speech. They also demonstrated that the effect of spectral degradation on semantic processing involving expectation and context was more pronounced in ordinary speech than in vocoded speech. However, such congruent/incongruent semantic paradigms in sentences come with the limitation of being dependent on contextual cues, making it challenging to control for individuals’ education and cognitive abilities. It has been acknowledged that humans rely more on top-down processing when the spectral information in the speech signal is degraded (Shannon et al., 1995; Davis et al., 2005; Obleser and Eisner, 2009; Peelle and Davis, 2012).

To explore the impact of vocoder channel count on semantic processing without contextual cues, we utilized a one-syllable oddball paradigm, and measured the N2 and P3b components associated with purely acoustic-based semantic processing. Based on our results, we suggest that the N2 and P3b responses induced by a one-syllable task generated using a vocoder may serve as suitable objective measures for representing spectral degradation as a function of the number of channel bands. The earlier cortical potentials, between 100 and 200 ms after the stimulus, are more related to the bottom-up perception of speech, and there is a possibility of a robust response even if the meaning of spectrally degraded speech cannot be understood. Therefore, the impact of spectral degradation in a noise vocoder could be more closely related to the semantic system than to the perception itself, especially when there is minimal redundancy in the available cues. Collectively, our results suggest that N2 and P3b responses, measuring the top-down mechanism of speech comprehension, would be useful tools for representing the effects of the number of channel bands.

Several studies have examined the neural representation of vocoded speech at the brainstem level. Using the frequency following responses (FFR), for example, neural facilitation was intimated as a function of the number of channel bands (Ananthakrishnan et al., 2017) reported that, using FFR, the improvement in brainstem F0 magnitudes, phase-locked to the temporal envelope of the stimuli as the number of channels increased from 1 to 4 consistent with the behavioral performance. However, the F0 representation was followed by a plateau with 8 and 16 channels and then a degradation with 32 channels. Using FFR and cortical auditory-evoked potentials (Kong et al., 2015), confirmed that attention modulates EEG entrainment to the speech envelope and that the neural entrainment measured using correlation coefficients between the N1 response and speech envelope increased as a function of the number of channel bands.

A limitation of this study was the relatively small size of the study sample. However, we endeavored to control the age of the participants along with their duration of education and attention level throughout the tests. Because we controlled the age of the listeners (young participants), we could not generalize our conclusion to a wider population, such as the elderly, in the current study. Another limitation is that in the analysis of the EEG response, both correct and incorrect responses were used without excluding the incorrect ones due to the very low correct response rate. Although excluding trials with an incorrect behavioral response could ensure a more accurate interpretation of the EEG response, we were concerned that deleting too much data would decrease the reliability of the overall dataset. Future research could consider expanding participants to the various age groups. Furthermore, it would be necessary to conduct studies measuring cortical responses in listeners with hearing impairment characterized by sparse spectral resolution.

Our data enhance understanding of how spectral information influences cortical speech processing, and they have implications for developing advanced algorithms in hearing aids for individuals with hearing impairments or degraded auditory input. Moreover, a better understanding of the impact of sparse spectral information on speech intelligibility and neural representation could provide valuable insights for designing public places such as auditoriums or transportation hubs.

## 5 Conclusion

Our study has demonstrated that the degree of spectral richness plays a crucial role in both speech perception and neural responses. We particularly adopted a one-syllable paradigm to elicit N2 and P3b responses, a higher-order cognitive process, while minimizing contextual cues and controlling the education and the attention level across participants. The N2 and P3b responses proved to be highly sensitive to the effects of spectral degradation, surpassing its sensitivity to speech perception.

## Data availability statement

The original contributions presented in the study are included in the article/supplementary material, further inquiries can be directed to the corresponding author.

## Ethics statement

The studies involving humans were approved by Review Board of Nowon Eulji Medical Center. The studies were conducted in accordance with the local legislation and institutional requirements. The participants provided their written informed consent to participate in this study.

## Author contributions

HC: Methodology, Writing – original draft, Data curation, Investigation. J-SK: Data curation, Formal analysis, Methodology, Writing – original draft, Writing – review & editing. JW: Methodology, Writing – review & editing. HS: Conceptualization, Funding acquisition, Methodology, Supervision, Writing – original draft, Writing – review & editing.
